# A Wireless and Portable Electronic Nose to Differentiate Musts of Different Ripeness Degree and Grape Varieties

**DOI:** 10.3390/s150408429

**Published:** 2015-04-13

**Authors:** Manuel Aleixandre, Jose Pedro Santos, Isabel Sayago, Juan Mariano Cabellos, Teresa Arroyo, Maria Carmen Horrillo

**Affiliations:** 1 GRIDSEN, Instituto de Tecnologías Físicas y de la Información (ITEFI-CSIC), Madrid 28006, Spain; E-Mails: manuel.aleixandre@gmail.com (M.A.); jp.santos@csic.es (J.P.S.); i.sayago@csic.es (I.S.); 2 Dpto. Investigación Agroalimentaria, Instituto Madrileño de Investigación y Desarrollo Rural, Agrario y Alimentario (IMIDRA), Madrid 28800, Spain; E-Mails: juanmariano.cabelloscaballero@gmail.com (J.M.C.); teresa.arroyo@madrid.org (T.A.)

**Keywords:** electronic nose, degree of ripeness, must, analytical parameters, PCA, PNN, CCA

## Abstract

Two novel applications using a portable and wireless sensor system (e-nose) for the wine producing industry—The recognition and classification of musts coming from different grape ripening times and from different grape varieties—Are reported in this paper. These applications are very interesting because a lot of varieties of grapes produce musts with low and similar aromatic intensities so they are very difficult to distinguish using a sensory panel. Therefore the system could be used to monitor the ripening evolution of the different types of grapes and to assess some useful characteristics, such as the identification of the grape variety origin and to prediction of the wine quality. Ripening grade of collected samples have been also evaluated by classical analytical techniques, measuring physicochemical parameters, such as, pH, Brix, Total Acidity (TA) and Probable Grade Alcoholic (PGA). The measurements were carried out for two different harvests, using different red (Barbera, Petit Verdot, Tempranillo, and Touriga) and white (Malvar, Malvasía, Chenin Blanc, and Sauvignon Blanc) grape musts coming from the experimental cellar of the IMIDRA at Madrid. Principal Component Analysis (PCA) and Probabilistic Neural Networks (PNN) have been used to analyse the obtained data by e-nose. In addition, and the Canonical Correlation Analysis (CCA) method has been carried out to correlate the results obtained by both technologies.

## 1. Introduction

The wine industry generates millions of dollars in sales annually and there is much new and interesting research currently being done to promote wine quality. Grape control and monitoring are very important since grape quality at harvest is the main factor that conditions future wine quality [1]. There are extensive studies on wine regarding the vinification process and the final product (measuring defects, aromatic qualities, *etc.*). In these studies though the musts, grape juice prior to fermenting, are mostly ignored.

The composition of grapes at the time of picking is an important parameter during wine production, which may be considered the most crucial in winemaking. While grapes ripen, some complex physicochemical and biochemical processes occur, such as the continuous rising and lowering of sugar concentrations and acid levels, respectively, and the increase and evolution of aromatic compounds (terpenes, norisoprenoids, benzene compounds and alcohols), which are influenced not only by the cultivar but also by genetic, climatic and geographical factors and by cultivation practices [2,3].

The factors defining the ripeness process determine grape quality and the optimal time of harvesting for winemaking. Traditionally, harvesting date indicators have been determined by parameters such as berry weight or must density. Nowadays, Near Infrared Spectroscopy (NIRS) has also been reported as a suitable technique capable of measuring parameters such as sugar content, pH value and acidity in grapes, but it requires expensive instrumentation and complex calibration [4]. In fact, even one of the simplest traditional systems to determine harvesting date, *i.e.*, the determination of sugar content (°Brix) and Total Acidity, requires the use of a refractometer and a titration system, which employs chemical products [5]. Therefore, the possibility of designing simple, rapid and of low cost alternative methods to monitor grape ripeness is of great interest. It is necessary to find a fast and cheap method in contrast with the conventional analytical methods to analyse the volatile compounds of musts.

An electronic nose is a device that produces a signal that when properly calibrated can be correlated with the composition of an aromatic/gaseous mixture. There are many electronic noses capable of identifying and classifying food samples (through the headspace method), and of quantifying the concentration of particular volatile compounds or organoleptic attributes [6]. Therefore, the aroma composition of berry destined for winemaking may provide useful information for predicting wine quality [7].

Electronic noses are usually composed of an array of chemical sensors coupled with a pattern recognition analysis. Another aim in electronic nose development is to design easy-to-use reduced-in-size systems applicable for *in-situ* and on-line monitoring. Besides, attempts have also been made to correlate electronic nose data with traditional human sensory perceptions of wine attributes, and with gas chromatography-mass spectrometry results [8,9,10,11]. Few studies on the potential use of electronic noses for grape ripeness monitoring, converted to musts, have been reported [12,13,14,15,16], and none comparing the results to a sensory analysis. This is surely due to the similar and low aromatic intensity of musts that making it difficult to distinguish by a tasting panel.

Following our interest in the development of sensing systems and given our experience in designing electronic nose devices for wine applications [17,18,19,20,21,22,23,24], we report herein the design and development of an e-nose, realized in our laboratory, as an useful tool for this kind of analysis. In this work a wireless and portable e-nose (WiNOSE 2.0) has been used to monitor the volatile organic compounds (VOCs) of musts of different grape varieties and different grades of ripeness for several harvests, and to relate its responses with the physicochemical parameters which are traditionally used to determine the harvesting date.

## 2. Experimental Section

### 2.1. Samples Measured

Musts of eight different grape varieties: four white ones (Chenin Blanc, Sauvignon Blanc, Malvar and Malvasia) and four red ones (Tempranillo, Barbera, Touriga and Petit Verdot) and with different grape ripening times, have been measured. Table 1 and Table 2 show the dates of the samples for each variety, used for the measurements of the physicochemical parameters and for the measurements of the electronic nose respectively. All these grape varieties were grown in the IMIDRA (Madrid, Spain) during the years 2011 and 2012. More details of these varieties are given in [25,26].

**Table 1:** Grape collection date of the different varieties used for the physico-chemical parameter measurements.

Grape Varieties	Month-Day	Year
Barbera	8-16, 8-22, 8-16, 8-29, 9-12	2011
Petit Verdot	8-22, 8-29, 9-5, 9-12, 9-19	2011
Tempranillo	8-11, 8-16, 8-22, 8-29, 9-5, 9-12	2011
Touriga	8-16, 8-22, 8-29, 9-5, 9-12	2011
Malvar	8-16, 8-22, 8-29, 9-1	2011
Malvasía	8-16, 8-22, 8-29, 9-6	2011
Chenin Blanc	8-16, 8-22, 8-29, 9-5, 9-12	2011
Sauvignon Blanc	8-11, 8-16, 8-22	2011
Barbera	8-21, 9-10, 9-17, 9-25	2012
Petit Verdot	9-3, 9-10, 9-17, 9-24	2012
Tempranillo	8-14, 8-21, 8-27, 9-3	2012
Touriga	8-21, 8-27, 9-3, 9-10, 9-17	2012
Malvar	8-14, 8-21, 8-27, 9-3, 9-12	2012
Malvasia	8-14, 8-21, 8-27, 8-30	2012
Chenin Blanc	8-28, 9-3, 9-10	2012
Sauvignon Blanc	8-14, 8-21, 8-27, 9-5	2012

**Table 2:** Grape collection date of the different varieties used for the electronic nose measurements.

Grape Varieties	Month-Day	Year
Barbera	8-16, 8-22, 9-5, 9-14	2011
Petit Verdot	8-22, 9-12, 9-22	2011
Tempranillo	8-16, 8-22, 9-5, 9-14	2011
Touriga	8-16, 8-28, 9-5, 9-14	2011
Malvar	8-16, 8-22, 9-1	2011
Malvasía	8-16, 8-22, 9-6	2011
Chenin Blanc	8-16, 8-22, 9-5, 9-12	2011
Sauvignon Blanc	8-16, 8-22, 9-25	2011
Barbera	8-21, 9-10, 9-17, 9-25	2012
Petit Verdot	9-10, 9-18, 9-24	2012
Tempranillo	8-14, 9-3	2012
Touriga	8-21, 9-10, 9-17	2012
Malvar	8-14, 9-3	2012
Malvasia	8-14, 8-31	2012
Chenin Blanc	9-3, 9-10	2012
Sauvignon Blanc	8-14, 9-4	2012

The grape sampling in field was done by collecting 3–4 bunches on 100 strain berries, alternating bunches shaded and exposed to light, at different heights on the vines and on bunches up to 1.5–2 kg, approximately between 1000–2000 berries. Then, they were crushed and centrifuged at a controlled temperature (10 °C) to obtain the musts.

### 2.2. Physicochemical Parameters

Different characteristic chemical and physical parameters of the grapes of these musts have been measured. Grade Brix (Bx), percentage of sucrose dissolved in the must, was measured by refractometry (Ataio PR-100). Probable alcoholic grade (PAG) is calculated by the approximation of dividing the sugar concentration (SU) in gr·L^−1^ by 17 (being 17 the amount of sugar the yeast needs to make an alcoholic grade). pH and Total Acidity (TA) in gr·L^−1^ of tartaric acid by potenciometry through a Crison Compact Titrator. Technology maturity index (TMI) is calculated by SU/TA, weight of 100 berries (W100) and number of berries in 100 grams (#100B).

### 2.3. System of Measurement with Electronic Nose

**Figure 1:**
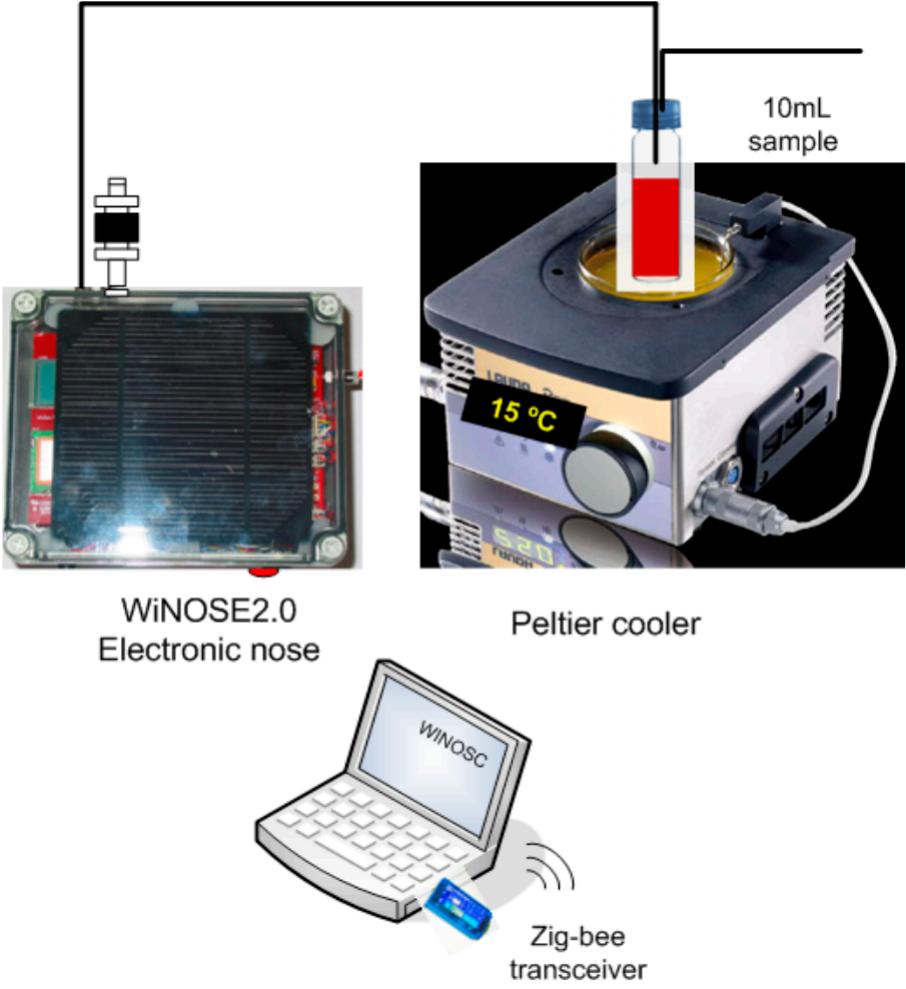
Experimental system of measurement.

The measurement system is displayed in Figure 1 and is formed by: (1) Volatile organic compound extraction method; (2) Peltier cooler; (3) WiNOSE 2.0 with a resistive sensor array and control system.

(1) Volatile organic compound extraction method

The extraction method used is head space with dynamic injection of the volatile compounds onto the multisensor using air as carrier gas.

(2) Peltier cooler

To keep the sample temperature at 15 °C and thus to minimise the oxidation of the compounds a Peltier system is used.

(3) WINOSE 2.0 with a resistive sensor array

The core of the electronic nose is a commercial MSGS-4000 microsensor array (Silsens, Newchatel, Switzerland). It consists of four thin nanocrystalline tin oxide layers deposited over micromechanised silicon hot plates. One of the microsensors is doped with platinum. Every individual sensor operates at a temperature between 200 °C and 350 °C.

The whole system is controlled by a digital signal controller (model dsPIC33FJ128GP306, DSC Microchip, Chandler, AZ, USA). It is a 16 bit microcontroller with 16 Kb of RAM and 128 Kb of FLASH memory. It has several analogue to digital converter (A/D) inputs for sensor measurements and several pulse width modulation (PWM) outputs for sensor heating. The main measurement parameters are shown in a LCD. Wireless communications are provided by a ZigBee IEEE 802.15.4 transceiver. Beneath the electronic board two 4500 mA·h batteries are placed. The instrumentation is controlled by a programme developed in Testpoint. See details in [27].

One of the gas inlets has a carbon filter to provide clean air as a reference baseline. The humidity and temperature sensors (SHT15, Sensirion, Staefa, Switzerland), the pump (model 2002, Rietschle Thomas, Fürstenfeldbruck, Germany) and the flowmeter (PFMV5, SMC, Tokyo, Japan) are located downstream.

### 2.4. Measurement Procedure

The bottles with the must samples were frozen as they were taken and then defrosted for 15 min. before starting the measurement process. 10 mL of must were put into vials and kept at 15 °C. To transfer the headspace to the sensors the flow of air was set at 55 mL/min during the sampling and at 200 mL/min during the recovery. The sampling and recovery times were 1 and 14 min, respectively, to reach equilibrium. Resistance and other operating parameters (humidity, flow and temperature) were measured every two seconds. The system showed an excellent response, reproducibility and stability.

### 2.5. Data Treatment

The response of the sensor is calculated by dividing the resistance of the sensor after the vapour exposure by the resistance of the sensor in air. The measurement process consisted of the repetition of ten measurements in succession. Several of these measurements were made with different aliquots in order to check for repeatability.

Principal component analysis (PCA) is a statistical method for reducing the number of dimensions of numerical dataset. Mathematically, PCA projects the data onto a new coordinate base formed by orthogonal directions, which contains a growing amount of the variance of the data. The principal components are ordered, thus the greatest variance is on the first coordinate (called first principal component, PC1), the second greatest variance is on the second coordinate, PC2, and so on. PC1, PC2 and PC3 allow the visualisation of dataset main information in 2-D and 3-D representation.

A probabilistic neural network (PNN) was applied to the PC1, PC2 and PC3 in order to recognize the type of VOCs patterns under study. Neural networks are mathematical models that process information by means of an adaptive system that changes its structure based on external or internal information that flows through the network during the learning phase. Thus, a neural network creates a function to capture and represent complex input/output relationships. The PNN is a type of neural network with radial basis transfer functions that measures the distance between an input vector and the training vectors. A PNN was trained, and the performance was evaluated with the leave-one-out validation method [28]. This method consists of training N distinct nets (in this case, N is the number of the measurements) with all the data minus a vector excluded from the training set but used as a validation test This procedure is repeated N times until all the vectors are validated.

Canonical correlation analysis consists of a correlation of two groups of variables. The method searches linear combinations on one group that best correlates with a linear correlation on the other group. To do this it selects an axis in each group that maximises the variance in that group while it tries to maximise the amount of covariation between the two variable groups. High correlations mean that both groups share common information and it is usually measured by the r coefficient and the assessment of statistical significance with Wilk’s lambda test. The coefficients of each linear combination give us information of what variables are correlated. A high coefficient on a variable marks that variable as correlated with the other group while a low coefficient (in absolute value) on a variable means a lower correlation.

## 3. Results and Discussion

**Figure 2:**
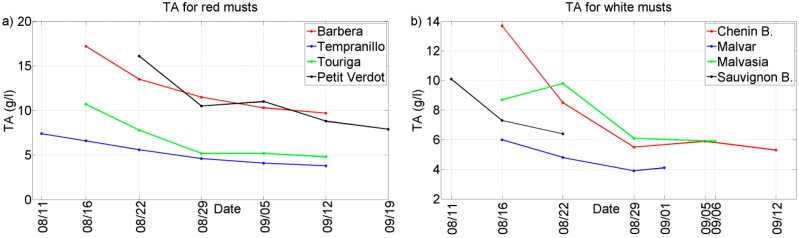
Evolution of TA for musts of 2011 harvest. (**a**) Red musts; (**b**) White musts.

**Figure 3:**
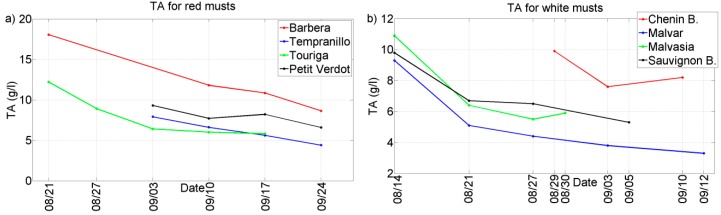
Evolution of TA for musts of 2012 harvest. (**a**) Red musts; (**b**) White musts.

**Figure 4:**
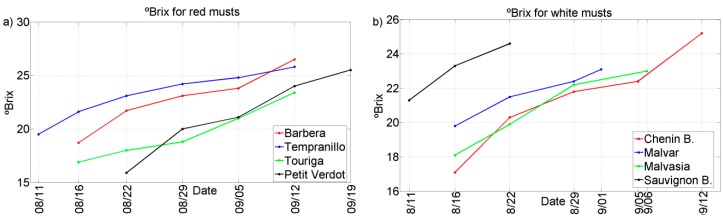
Evolution of ºBrix for musts of 2011 harvest. (**a**) Red musts; (**b**) White musts.

**Figure 5:**
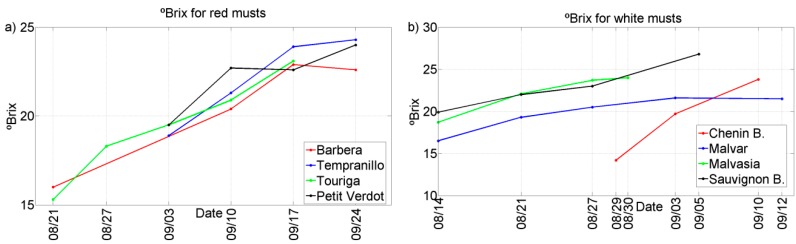
Evolution of °Brix for musts of 2012 harvest. (**a**) Red musts; (**b**) White musts.

Two representative chemical parameters (°Bx and TA) have been plotted against the different grape ripening stages for each of the grape varieties collected during the harvests of 2011 and 2012. See Figure 2, Figure 3, Figure 4 and Figure 5. In general, we can observe that these parameters follow the typical trend of ripening in terms of the physiological ripeness of grapes, *i.e.*, an increase in the *sugar content and a decrease in the total acidity*, showing the evolution of the musts with the ripening time.

**Figure 6:**
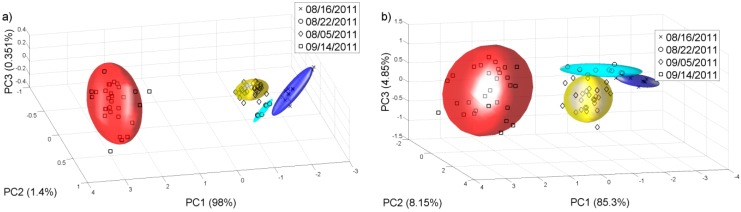
PCA plot for different grades of grape ripening in 2011. (**a**) Chenin Blanc; (**b**) Barbera.

Figure 6 shows the PCA plot for different grades of grape ripening for 2011 must samples of Chenin Blanc and Barbera. This data analysis shows that the e-nose can differentiate the different grape ripening grades for different grape varieties. It is important to note that one of the stages that more influences the wine aromatic characteristics of a given variety is the ripening of the grape, since both free and glycosylated forms of varietal compounds accumulate in the grape during ripening [29]. Terpene content may decrease once optimal sugar levels are attained, although this may be influenced by temperature and water availability during ripening [30]. For the samples collected in September, three different aliquots have been taken in order to see if the results were reproducible. In this case 30 measurements were realised (Figure 6).

**Figure 7:**
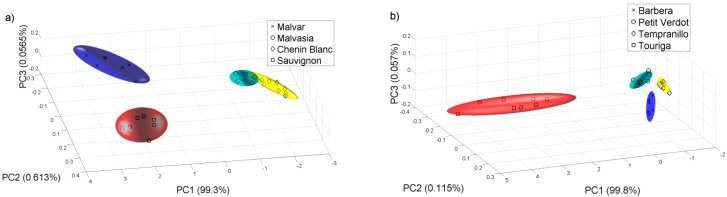
PCA analysis for the samples collected on 22 of August of 2011. (**a**) White varieties; (**b**) Red varieties.

Then we did another PCA analysis for all the samples collected on 22 of August of 2011. Figure 7 shows the PCA plot of the different grape varieties. We check that the e-nose can differentiate the varieties of white and red grape musts even at this early stage of the grape ripening.

**Figure 8:**
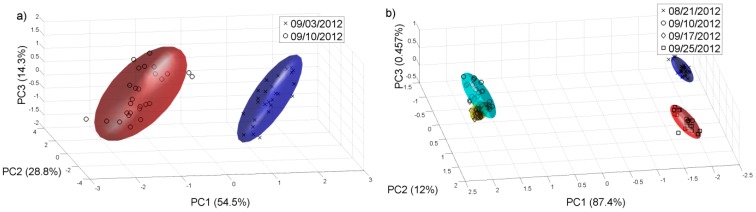
PCA plot for different grades of grape ripening in 2012. (**a**) Chenin Blanc; (**b**) Barbera.

**Figure 9:**
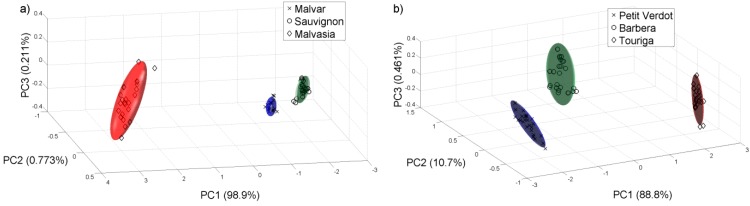
PCA analysis for the samples collected in 2012. (**a**) 14 of September for white varieties; (**b**) 10 of September for red varieties.

We repeated the same analysis for the year 2012 with the samples available. Figure 8 and Figure 9 show the PCAs carried out for this year, and it is possible to observe that for the harvest 2012, the e-nose can also discriminate the different grape ripening grades for the different white and red varieties and the varieties of white and red grape musts for the samples collected on 14 and 10 of September respectively, in this case, for a late stage of the grape ripening. The Probabilistic Neural Network (PNN) validated by the leave-one-out method gives a good ripening grape classification for each variety from the 2011 harvest, showing that the ripening process can be detected by the sensor system. See Table 3. The PNN analysis was repeated for all the samples collected on 22 of August 2011. The classification of the validated PNN (red varieties 98.1% and white varieties 93.6%) shows that there is a clear differentiation, and therefore the e-nose system could be used to assess some of the characteristics corresponding to the different varieties.

**Table 3:** Classification by a PNN for samples collected in 2011.

Red Grape Varieties	Classification	White Grape Varieties	Classification
Barbera	98.8%	Malvar	95.5%
Petit Verdot	85.5%	Malvasía	98.0%
Tempranillo	98.9%	Chenin Blanc	94.8%
Touriga	99.1%	Sauvignon Blanc	92.5%

Again by repeating the process for the 2012 harvest, similar results were obtained. The separation for the grape ripening grade is even better. The classification task is easier due to the lowest number of classes in the experiments. Table 4 summarises the results.

We also apply the PNN analysis to the samples collected on 10 and on 14 of September for different red and white varieties available. A 100% classification result was obtained for the white musts and a 98.7% was achieved for the red ones.

**Table 4:** Classification by a PNN for samples collected in 2012.

Red Grape Varieties	Classification	White Grape Varieties	Classification
Barbera	91.9%	Malvar	100%
Petit Verdot	99.7%	Malvasía	100%
Tempranillo	100%	Chenin Blanc	100%
Touriga	98.6%	Sauvignon Blanc	90%

**Figure 10:**
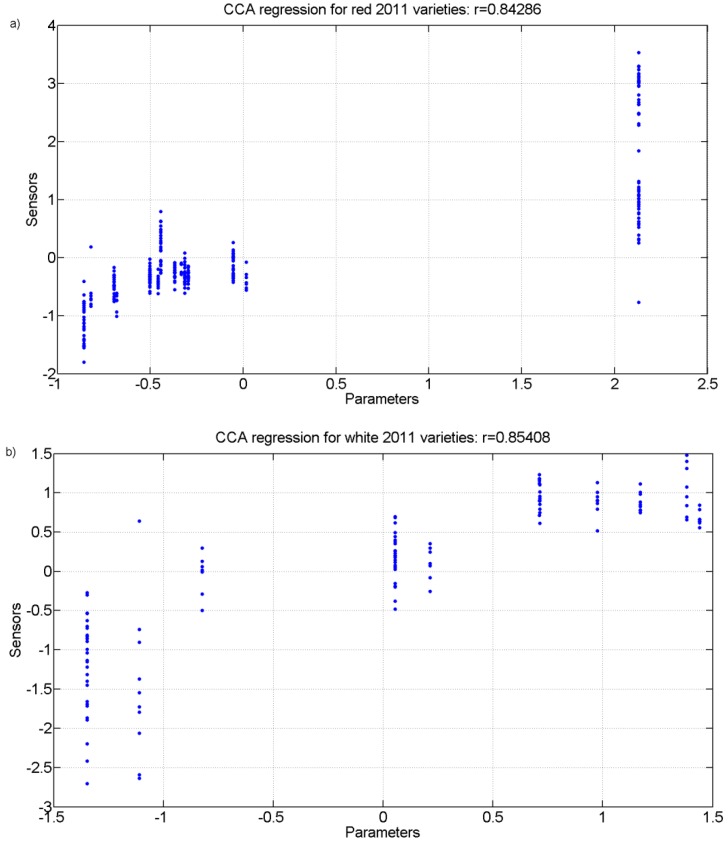
r values for the canonical correlation between grape must parameters and sensor responses for grape must varieties of 2011. (**a**) Red varieties; (**b**) White varieties.

**Figure 11:**
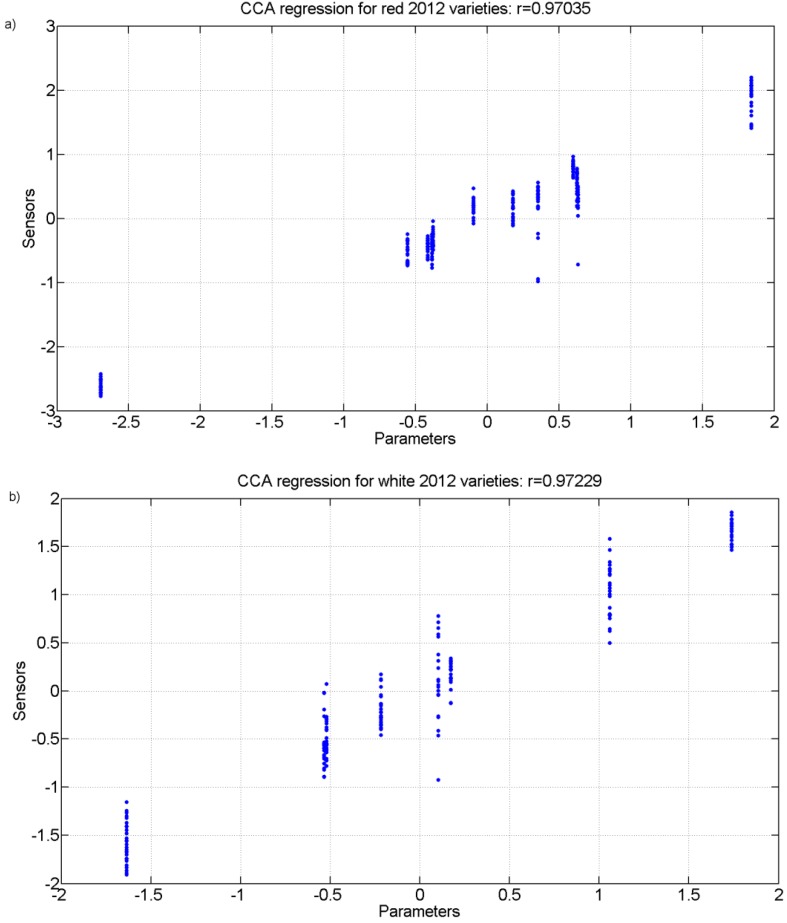
r values for the canonical correlation between grape must parameters and sensor responses for grape must varietiesof 2012. (**a**) Red varieties; (**b**) White varieties.

Finally, a Canonical Correlation Analysis (CCA) was carried out. Figure 10 and Figure 11 show the regression plot of this analysis for the white grape must and the red grape must sensor responses and all analytical parameter values measured for the 2011 harvest and 2012 harvest, respectively.

Table 5, Table 6, Table 7 and Table 8 show the correlation coefficients for the physicochemical parameters of interest for the white and red grapes for both harvests for up to four correlations found. The analysis and the r coefficients obtained show that there is a correlation between the sensor responses and some of the physicochemical parameters measured. There is also a statistical significance with Wilk’s lambda test that produced p values that are less than 0.01 for all correlations thus showing that the results are statistically significant. For the highest correlation CCA r values, the parameters of total acidity (TA) and pH had the greatest coefficients showing that they were the best correlated with the sensor responses.

**Table 5:** CCA coefficients of the first four canonical correlations for red musts of 2011.

Can.Correl. No.	r	Brix	pH	AT	Az g/L	IMT
1	0.84	0.11	‒4.90	‒1.31	‒0.02	0.43
2	0.74	0.37	1.98	‒1.53	0.04	‒0.14
3	0.40	0.83	‒9.76	6.09	‒0.20	0.87
4	0.15	0.98	‒2.97	9.23	0.40	0.25

**Table 6:** CCA coefficients of the first four canonical correlations for white musts of 2011.

Can.Correl. No.	r	Brix	pH	AT	Az g/L	IMT
1	0.85	0.11	3.67	‒0.30	‒0.16	‒0.36
2	0.66	0.40	0.07	‒0.36	‒0.07	‒0.06
3	0.51	0.69	6.50	‒7.52	0.24	‒0.52
4	0.26	0.93	‒1.22	3.79	1.13	0.05

**Table 7:** CCA coefficients of the first four canonical correlations for red musts of 2012.

Can.Correl. No.	r	Brix	pH	AT	Az g/L	IMT
1	0.97	0.00	‒0.17	‒3.07	0.09	0.01
2	0.93	0.03	‒0.03	1.28	‒0.13	0.04
3	0.84	0.24	‒0.17	4.65	‒0.18	0.02
4	0.46	0.79	‒0.98	‒1.27	‒0.49	0.04

**Table 8:** CCA coefficients of the first four canonical correlations for white musts of 2012.

Can.Correl. No.	r	Brix	pH	AT	Az g/L	IMT
1	0.97	0.01	‒2.89	6.23	0.34	0.22
2	0.89	0.11	‒2.52	7.67	1.47	0.21
3	0.69	0.52	1.87	‒20.53	1.63	‒0.14
4	0.10	0.99	‒0.52	0.30	0.79	0.07

The sensor signal depends on the aromatic composition of the must. Traditionally, to follow berry ripening, classical parameters based on the percentage of soluble solids, sugar, acidity, pH and colour are used [31]. However, in order to trace more specifically the varietal characteristics and to achieve a better product quality, an analysis of phenolic, carotenoids and volatile compounds should be carried out [32]. The existence of a correlation between the classical parameters and the response of the sensors is useful for the determination of must quality which is similar to the classic analysis method, but the electronic nose has also into account aromatic properties and, in addition, it is a faster, cheaper and simpler method.

## 4. Conclusions

In this work, it has been possible to demosntrate a complex and new application related to the grape musts which are of the utmost interest for the wine producing industry due to the fact there is a great relation between the grape ripening grade and wine quality. In this case, red and white grape variety musts with different grades of ripening, which cannot be discriminated easily by a sensory panel due to their similar and low aromatic intensity, have been identified through the measurement of must samples with a portable and wireless e-nose. The e-nose measurements for musts made with different grape ripening grades and different red and white varieties have been analysed by PCA and PNN showing that the e-nose can differentiate and classify the different ripening grades of the grape, converted in musts, when correctly trained. It has also been achieved the application of discriminating the different white (Malvar, Malvasía, Chenin Blanc, and Sauvignon Blanc) and red (Barbera, Petit Verdot, Tempranillo, and Touriga) grape varieties. For both applications, the results have been repeated for the samples from the harvest of 2011 and of 2012 collected during the August and September months. Therefore it is possible to concludethat the results obtained by the e-nose are consistent and repeatable.

Different physico-chemical parameters (°Bx, PGA, pH and TA) have been measured, for the same ripening samples tested by the e-nose, through traditional analytical methods used to monitor the changes of these parameters during the grape ripening process.

It is seen that both the analytical parameter obtained values and the PCA plots of the responses of sensors show a clear evolution with the ripening stages of the musts and are correlated as shown by the CCA analysis, mainly with the TA and pH. This correlation implies that there is information shared between the analytical parameters and sensor responses. In the same way, a commercial electronic nose was also used to discriminate grape ripening grades but, in this case, one unique variety of red grape (Cabernet Sauvignon) was tested [12], Hence the electronic nose technology is an alternative method to monitoring grape ripeness and, thereby to evaluate the optimal time to carry out harvesting in order to achieve a better quality wine.
